# Print Velocity Effects on Strain-Rate Sensitivity of Acrylonitrile-Butadiene-Styrene Using Material Extrusion Additive Manufacturing

**DOI:** 10.3390/polym13010149

**Published:** 2021-01-01

**Authors:** Wilco M. H. Verbeeten, Rob J. Arnold-Bik, Miriam Lorenzo-Bañuelos

**Affiliations:** 1 Structural Integrity Research Group, Universidad de Burgos, Avenida Cantabria s/n, E-09006 Burgos, Spain; mlbanuelos@ubu.es; 2 Department of Mechanical Engineering, Eindhoven University of Technology, P.O. Box 513, NL-5600 MB Eindhoven, The Netherlands; thatsmeuno@hotmail.com

**Keywords:** 3D printing, ABS, printing speed, strain-rate dependent yield stress, process-induced molecular orientation, Eyring rate equation, strain-dependent activation volume

## Abstract

The strain-rate sensitivity of the yield stress for Acrylonitrile-Butadiene-Styrene (ABS) tensile samples processed via material extrusion additive manufacturing (ME-AM) was investigated. Such specimens show molecular orientation and interstitial voids that affect the mechanical properties. Apparent densities were measured to compensate for the interstitial voids. Three different printing speeds were used to generate ME-AM tensile test samples with different molecular orientation. Printing velocities influenced molecular orientation and stretch, as determined from thermal shrinkage measurements. Likewise, infill velocity affected the strain-rate dependence of the yield stress. The ABS material manifests thermorheollogically simple behavior that can correctly be described by an Eyring flow rule. The changing activation volume, as a result of a varying print velocity, scales linearly with the molecular orientation, as captured in an estimated processing-induced pre-strain. Therefore, it is suggested that ME-AM processed ABS shows a deformation-dependent activation volume. This paper can be seen as initial work that can help to improve quantitative predictive numerical tools for ME-AM, taking into account the effects that the processing step has on the mechanical properties.

## 1. Introduction

Additive Manufacturing is a freeform fabrication technique that is rapidly gaining interest from industry, as well as increased research efforts from academic organizations. Its application is slowly shifting from Rapid Prototyping (RP) towards Rapid Manufacturing (RM) for the fabrication of customized end-use parts [1]. For polymer components, Material Extrusion Additive Manufacturing (ME-AM) [2], also known as Fused Deposition Modeling (FDM^®^), Fused Filament Fabrication (FFF), Fused Layer Modeling (FLM), or 3D printing, is one of the most prominent techniques. Its popularity derives from a combination of low investment costs, a wide variety of materials, and ease for manufacturing [3,4].

In Material Extrusion Additive Manufacturing equipment, the feedstock material is mostly added in the form of a thermoplastic polymer filament. A pinch roller mechanism pushes the filament through a heated liquefier, i.e., the zone where the material melts. The molten polymer is then further pushed and extruded through a heated nozzle. By depositing the molten material in a controlled manner onto a (heated) build platform or an already deposited and solidified layer, complex 3D objects can be constructed. After leaving the heated nozzle, the hot polymer bonds with the underlying surface through wetting and molecular diffusion driven by reptation [5,6,7], while it rapidly cools down and solidifies [3,8]. The resulting end-product is a laminate composite structure consisting of stacked layers of partially bonded filaments with interstitial voids [9,10].

During an ME-AM build cycle, material elements experience *local*, and therefore different, time-strain and time-temperature profiles [6,7,10,11,12,13,14], leading to a certain mesoscopic voided structure [8,15]. Hence, due to the particularity of the ME-AM process, a 3D printed component has a heterogeneous mechanical property distribution along its geometry [16,17], with both bulk-like (within a strand) as well as non-bulk-like (in strand-to-strand bonds) properties [12], which is influenced by the chosen AM processing parameters [8,9]. As a consequence, a certain macroscopic deformation behavior as measured in macroscopic tests (e.g., tensile, compression, shear, torsion, bending, creep, fatigue, impact) is the result.

Although *pseudo-isotropic* components can be obtained, e.g., by using a $0^{\circ }$/$90^{\circ }$/$+45^{\circ }$/$-45^{\circ }$ infill orientation stacking sequence [9,18], ME-AM technology produces parts with inherent *local* anisotropy. This is caused by two effects: (i) there is a difference in stiffness and strength in the direction of a strand (inter-strand strength) and perpendicular to the strand direction (intra-strand bond strength) [8,9,12,17]; (ii) the shear effects in the nozzle and the curvature between the nozzle and the deposited strand will lead to orientation and stretch of the polymer chain [6,13,19], thus leading to anisotropy within a single strand. Depending on printing speeds, shear rates can vary from under 100 1/s to upto 1000 1/s [14,20,21]. Furthermore, extrusion speed also affects the flow profile of the filament leaving the nozzle [22], and therefore influences orientation and stretch.

It was shown previously [9,17] that variations in molecular chain orientation and stretch produce different mechanical properties in ME-AM components. Additionally, macroscopic orientation, as induced by the strand infill orientation angle, generates distinctions in strain-rate dependence of the yield stress [17,23]. In addition, the strain-rate sensitivities manifested by the ME-AM samples was significantly different than that displayed by test specimen manufactured via compression molding using the same material [17].

Previous research on oriented polymers, as obtained by solid state forming processes such as hot drawing [24,25,26] or hydrostatic extrusion [24,27,28], also observed changes in strain-rate sensitivity. Using these forming processes, different degrees of pre-strain could be achieved by applying distinct draw ratios λ, ranging from λ=0 (i.e., iostropic material) to as high as λ=25 [24]. The maximum pre-deformation achieved for semi-crystalline polymers was significantly higher than that obtained for amorphous polymers [27]. High draw ratios of λ=25, equivalent to a pre-strain of $\varepsilon_{pre}=3.2$, could be obtained for semi-crystalline polymers [24], while moderate draw ratios upto λ=3.5, i.e., $\varepsilon_{pre}=1.25$, were achieved for an amorphous polymer [27]. By combining birefringence, yield stress, and shrinkage stress measurements, the degree of deformation (i.e., plastic pre-strain) was related to orientation and stretching of the molecular network [25,26]. Higher plastic pre-deformation (e.g., higher orientation) resulted in higher yield stresses [25,26] and higher strain-rate sensitivity [24,28]. The influence of strain on the strain-rate dependence of the stress, generally well-captured by an Eyring-type flow equation [29,30], was explained by one of the following two possibilities [24,28]: (i) a deformation-dependence of the activation volume, which provokes a gradual change in slope with pre-orientation; (ii) a deformation-dependence of the rate constant, which provokes a transition from the regime where only a single molecular process (α) is active to the regime with two active molecular relaxation processes (α+β).

Wendlandt et al. [31] looked at the large strain deformation behavior of several amorphous polymers. As strain increased, so did the strain-rate dependence of the measured stress. Hence, they suggested a strain-dependent activation volume, which was able to describe in good agreement the measured experimental data.

A similar investigation was performed on polycarbonate (PC) [32], and oriented polycarbonate and isotactic polypropylene (iPP) [33,34]. To obtain different degrees of orientation in PC, uniaxial tensile bars were pre-deformed to strain levels as high as $\varepsilon_{pre}=0.6$. Unoriented and oriented iPP tapes were accomplished by hot drawing to draw ratios from λ=1 (isotropic) to λ=6 (anisotropic). Both the yield stress as well as stresses in the strain-hardening regime displayed higher strain-rate sensitivity as pre-strain and strain increased, i.e., enhanced orientation and stretch of the molecular network. A deformation-dependence of the Eyring rate constant was found for PC, meaning that pre-orientation causes a transition from the α- into the (α+β)-regime. On the other hand, a deformation-dependence of the Eyring activation volume was encountered for isotactic polypropylene. It was also mentioned that there exists a possibility that both the rate constant and the activation volume change with deformation [34].

Analogous pre-deformation related changes of the yield stress strain-rate sensitivity was also seen for injection-molded polypropylene [35] and polyethylene [36], exhibiting deformation-dependent activation volumes. Anisotropy and a crystalline orientation morphology in injection molded plates was confirmed by optical micrographs and FTIR spectrometry [35].

Sweeney et al. [37,38] implemented a strain-dependent activation volume in a constitutive model to describe ultra-high-molecular-weight polyethylene (UHMWPE). Predictions of the axial and transverse strain were obtained that were consistent with the experimental observations in tensile and compressive behavior.

Verbeeten et al. [17] investigated mechanical properties of polylactide (PLA) samples processed via ME-AM using different printing parameters. Their results showed that a change in infill orientation angle modified the strain-rate dependence of the yield stresses. However, distinct printing velocities, shown to alter the amount of stretch of the polymer chains, had an effect on the level of yield stress, but did not significantly influence the slope of strain-rate dependence. This seems to indicate that infill orientation angle determines the activation volume, i.e., strain-rate sensitivity, and that stretching of the polymer chains only influences the level of yield stress. Hence, this was interpreted as a deformation-dependent Eyring rate constant [17], similar to what was observed for PC [34].

In the present paper, an alternative route is taken to investigate the influence of ME-AM processing on variations in strain-rate dependence, and how it is related to the orientation and stretch of the polymer molecules. An Acrylonitrile-Butadiene-Styrene (ABS) polymer, a highly elastic material, is processed via ME-AM at distinct printing velocities to invoke differences in polymer chain orientation and stretch. Only samples were all strands in all layers are aligned in the longitudinal sample direction (i.e., infill orientation angle $\alpha_{or}=0^{\circ }$) are characterized, as the polymer mainly orients itself in the deposition direction [19]. Strain-rate sensitivity is measured for ABS tensile test specimen and analyzed with an Eyring-type flow rule [39]. The objective is to quantify to what extent printing velocity influences yield stresses and strain-rate dependence of ABS material. Furthermore, results can be used to assess if ABS shows a strain-dependent activation volume or a strain-dependent rate constant.

Thus, this research paper can help in the development of quantitative predictive numerical tools for material extrusion additive manufacturing (ME-AM). The viscoelastic behavior of polymers, as manifested in the initial stress–strain behavior up to yield [40,41] and the strain-rate dependence of the yield stress [29,39,42,43], is paramount to predict an ME-AM component’s ultimate failure behavior. This is not only important in short-term experiments, such as uniaxial tensile or compression tests, but also has a direct relation to long-term behavior, such as creep and fatigue [44,45,46].

## 2. Material and Methods

### 2.1. Material

A commercially available natural Acrylonitrile-Butadiene-Styrene (ABS) filament (Smart Materials 3D Printing S.L., Alcalá la Real, Spain) was used for the present research. The filament had a nominal diameter of 1.75 mm and a specific gravity of 1.04 g/cm${}^{3}$, according to the filament producer. Nozzle and bed temperatures are recommended to lie in the range from 230–250 ${}^{\circ }$C and 80–100 ${}^{\circ }$C, respectively. The material has a acrylonitrile, butadiene, and styrene content of approximately 35 mol%, 15 mol%, and 50 mol%, respectively, as measured with an NMR technique. The glass transition temperature $T_{g}$ of the material was measured using DSC, applying a heating–cooling–heating sequence under nitrogen at a scanning rate of 10 K/min and isothermal periods of 2 min. This resulted in $T_{g}=113$ ${}^{\circ }$C, consistent with literature values [47,48]. All samples were fabricated from a single spool, and the filament was used as-received directly after opening the vacuum-sealed bag in which it was shipped.

ABS is an important engineering rubber-toughened thermoplastic co-polymer, consisting of a glassy Styrene-AcryloNitrile (S-AN) matrix in which rubber PolyButadiene (PB) particles, grafted with styrene and acrylonitrile, are distributed. It is used for various applications in the automotive, electronic, construction, consumer goods, and household appliances industries. ABS has good mechanical properties, excellent impact toughness, favorable chemical resistance and surface appearance, and high dimensional stability, all at a relatively low cost [47,48]. It dominates the market for engineering polymers, accounting for appoximately 40% of the total demand in 2018 [49].

### 2.2. Material Processing

**Figure 1:**
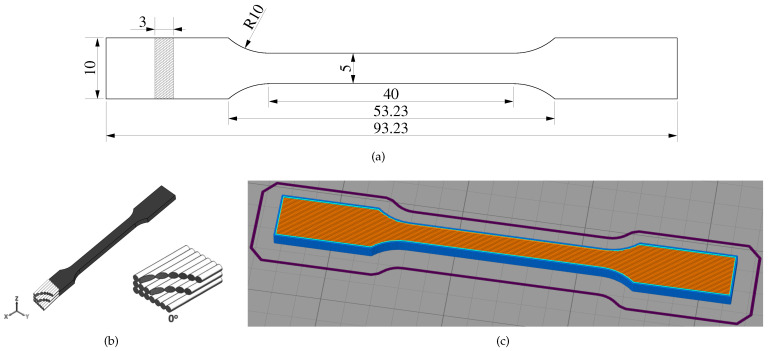
(**a**) tensile test specimen dimensions in mm; (**b**) schematic of fill pattern; (**c**) fill pattern configuration in Simplify3D.

Tensile samples were manufactured on an open-source RepRap Sirius 3D printer (Moebyus Machines, Madrid, Spain) using a 0.4 mm nozzle size. Sample dimensions are given in Figure 1a. This tensile test specimen is based on specimen type 1BA according to the ISO 527-2 norm, but adapted to avoid fracture in the fillet [17].

An STL-file of the tensile sample was imported in the Simplify3D slicing software in order to generate G-code files that were handled by the Sirius 3D printer. The infill orientation parameter was chosen to manufacture samples with a single strand infill orientation angle ($\alpha_{or}=0^{\circ }$), where all strands in all layers are aligned in the longitudinal sample direction (see Figure 1b). An example of the fill pattern configuration as generated by the Simplify3D software is given in Figure 1c. Three different printing speeds were chosen to generate samples with differences in polymer chain orientation and stretch. The complete set of printer parameters is given in Table 1. Once a test sample for a single printing velocity was correctly configured in the Simplify3D slicing software, it was copied 18 times and distributed in an equal manner over the surface representing the printer’s XY plane. For every printing velocity, a different G-code file was generated that manufactured 18 equivalent samples in a single print. These three G-code files were checked and only differed in printing speed, while the rest of the G-code stayed exactly the same, thus making sure that the other processing parameters were unaltered.

### 2.3. Mechanical Characterization

Uniaxial tensile tests were conducted at room temperature (23 ${}^{\circ }$C) on a MTS Criterion C43.104 universal test system, equipped with a 10 kN load cell. Constant linear strain rates in the range from $10^{-5}\;1/s$ to $10^{-1}\;1/s$ were applied for these tensile tests. At each strain rate, three samples were measured. As is common for polymers, the maximum in stress before softening in the stress–strain curve is treated as the material’s yield stress. True yield stresses are calculated from engineering values by assuming that the material’s volume remains constant during uniaxial tensile tests [30,50], at least up to yield stress.

### 2.4. Apparent Density

As ME-AM components generally present interstitial voids [9,10], their mechanical properties are influenced by this voided mesostructure. To compensate for these voids and provide a more fair comparison of the behavior of ME-AM processed samples, apparent densities were determined before performing any mechanical characterization tests. For every single sample, external dimensions were measured using a digital Mitutoyo micrometer and caliper (Mitutoyo Europe GmbH, Neuss, Germany). From these dimensions, the external nominal volume was calculated. Sample masses were measured using a precision Secura laboratory balance (Sartorius Lab Instruments GmbH & Co. KG, Goettingen, Germany). Apparent densities were computed from these values by applying:

(1) $$\rho_{app}=\frac{m_{sample}}{V_{sample}}\;.$$

Furthermore, an approximation of the porosity of the samples (in percentage) was determined by using the material reference density as given by the filament producer, i.e., $\rho_{ref}=1.04$ g/cm${}^{3}$:

(2) $$\mathrm{Porosity}=\frac{\rho_{ref}-\rho_{app}}{\rho_{ref}}\;.$$

Thus, a *void corrected* yield stress $\sigma_{y,vc}$ can then be calculated from the measured yield stress $\sigma_{y}$ by using the apparent density of each sample and the material reference density:

(3) $$\sigma_{y,vc}=\sigma_{y}\cdot \frac{\rho_{ref}}{\rho_{app}}\;.$$

### 2.5. SEM Fractography

Following uniaxial tensile tests, the fracture surface of several samples produced with the three different printing velocities were observed using an FEI Quanta 600 environmental scanning electron microscope (ESEM) (FEI Company Inc., Hillsboro, OR, USA). Samples were observed under vacuum and an accelerating voltage of 20.00 kV.

### 2.6. Polymer Chain Orientation and Stretch

To assess the extent of orientation and stretch of polymer chains during the ME-AM process, a qualitative macroscopic measurement procedure is applied that is based on thermal shrinkage [9]. By heating ME-AM processed samples above their glass transition temperature ($T_{g}=113$ ${}^{\circ }$C), oriented and stretched polymer chains will be able to relax and, as a consequence, samples will become shorter and wider. The relative shortening is a measure of the orientation in the sample, as indicated by Rodríguez et al. [9].

External sample dimensions of ME-AM test samples, processed at different speeds, are measured at room temperature using a digital Mitutoyo micrometer and caliper (Mitutoyo Europe GmbH, Neuss, Germany). Then, the samples are heated in an oven at 130 ${}^{\circ }$C for over 6 h. Next, the samples are cooled to room temperature and the external dimensions are measured again. Expansion(+)/contraction(−) percentages are determined from the dimensional differences. At least three samples for each printing velocity are used to determine these dimensional changes. Separate ME-AM processed ABS samples were used for either thermal shrinkage measurements or mechanical characterization. Additionally, thermal shrinkage measurements are also performed on the as-received filament.

### 2.7. Modeling

The deformation kinetics of polymers can be adequately characterized by a linear dependence of the yield stress on the logarithm of strain rate, on temperature, and on pressure [29,30,51]. An Eyring-type flow equation captures this behavior accurately:

(4) $$\dot{\bar{\gamma }}\left(T,\bar{\tau },p\right)={\dot{\gamma }}_{0}\exp\left(-\frac{\Delta U}{RT}\right)\exp\left(-\frac{\mu pV^{*}}{kT}\right)\sinh\left(\frac{\bar{\tau }V^{*}}{kT}\right)\;.$$

Here, $\dot{\bar{\gamma }}$ is the equivalent (Von Mises) plastic shear rate, ${\dot{\gamma }}_{0}$ a rate constant, ΔU the activation energy ($257\;kJ\;mol^{-1}$, as taken from literature [52]), *R* the universal gas constant ($8.314472\;J\;mol^{-1}\;K^{-1}$), *T* the absolute temperature in *K*, μ is a dimensionless pressure dependence parameter, *p* the hydrostatic pressure, $V^{*}$ the activation volume, *k* is the Boltzmann’s constant ($1.38054\times 10^{-23}\;J\;K^{-1}$), and $\bar{\tau }$ is the equivalent (Von Mises) shear stress.

Over time, Eyring’s flow rule has proven to be able to correctly describe the polymer’s thermally- and stress-activated plastic deformation mechanisms. The prefix ${\dot{\gamma }}_{0}$ is a rate constant that depends on the thermodynamic state of the material and is related to physical aging [53]. The first exponential term, which includes the activation energy ΔU, covers the material’s temperature dependence. It is related to the potential energy barrier that needs to be exceeded for segmental motion (i.e., molecular conformational changes). The last term, a hyperbolic sine function that includes the activation volume $V^{*}$, determines the stress dependency of the material. The stress in combination with the activation volume regulates the decrease of the potential energy barrier for segmental motion in the direction of the applied stress [31]. $V^{*}$ can be interpreted as a volume that is involved in a plastic deformation mechanism, and is related to the size of several statistical random links in the polymer chain that move simultaneously in a cooperative way [42,51]. The second exponential term, which includes the pressure dependence parameter μ, captures the effect of hydrostatic pressure. A negative hydrostatic pressure, e.g., as encountered in a tensile test experiment, lowers the potential energy barrier for molecular conformational changes. On the contrary, a positive hydrostatic pressure (e.g., during compression tests) increases this potential energy barrier. Therefore, Equation (4) can be used to evaluate (in a macroscopic sense) the potential energy barriers involved in the plastic deformation mechanisms at the yield stress, and the effects of temperature, strain rate, and pressure on those energy barriers.

For isotropic polymer materials, ΔU, $V^{*}$, and μ are generally considered to be intrinsic material constants. However, it was convincingly shown by several research groups [28,31,34] that the activation volume $V^{*}$ depends on molecular chain orientation and can be considered strain-dependent for certain polymers.

In this study, only tensile tests at room temperature and atmospheric pressure are performed. Therefore, the equation can be simplified. For uniaxial tensile tests, the equivalent plastic shear rate $\dot{\bar{\gamma }}$ and equivalent shear stress $\bar{\tau }$ (according to a Von Mises yield criterion) can be defined as:

(5) $$\dot{\bar{\gamma }}=\sqrt{3}\dot{\varepsilon }\;;\;\bar{\tau }=\frac{\sigma }{\sqrt{3}}\;.$$

Thus, if written in terms of the yield stress as a function of strain rate, the equation becomes:

(6) $$\sigma_{y}\left(\dot{\varepsilon }\right)=\frac{\sqrt{3}\;k\;T}{V^{*}}\sinh^{-1}\left[\frac{\sqrt{3}\;\dot{\varepsilon }}{{\dot{\gamma }}_{0}}\right]\;.$$

Thus far, measurements on ABS materials have only shown thermorheologically simple behavior [52,54,55,56]. However, in order to be able to determine if ABS material shows a strain-dependent rate constant, leading to a transition from the α- into the (α+β)-regime (similar to PC [34], and possibly ME-AM processed PLA [17]), the flow equation must be adapted to include thermorheologically complex behavior. For that, it is assumed that at least two molecular deformation processes govern the yield kinetics that act independently and in parallel [29,30,39]. The flow equation then reads:

(7) $$\sigma_{y}\left(\dot{\varepsilon }\right)=\sigma_{y,\alpha }\left(\dot{\varepsilon }\right)+\sigma_{y,\beta }\left(\dot{\varepsilon }\right)=\frac{\sqrt{3}\;k\;T}{V_{\alpha }^{*}}\sinh^{-1}\left[\frac{\sqrt{3}\;\dot{\varepsilon }}{{\dot{\gamma }}_{0,\alpha }}\right]+\frac{\sqrt{3}\;k\;T}{V_{\beta }^{*}}\sinh^{-1}\left[\frac{\sqrt{3}\;\dot{\varepsilon }}{{\dot{\gamma }}_{0,\beta }}\right]\;.$$

Note that true stress values are referred to in these previous equations.

## 3. Results and Discussion

First, the mechanical properties of the ME-AM ABS samples will be discussed, together with sample porosity and apparent density. Second, an Analysis of Variance is applied to the experimental data set in order to check its consistency. Then, the polymer chain orientation and stretch are analyzed as affected by the print velocity. Next, it is discussed how these experimental results are related to the activation volume as present in Eyring’s flow rule. Finally, SEM fractography images of the ABS samples will be analyzed.

### 3.1. Mechanical Characterization

**Figure 2:**
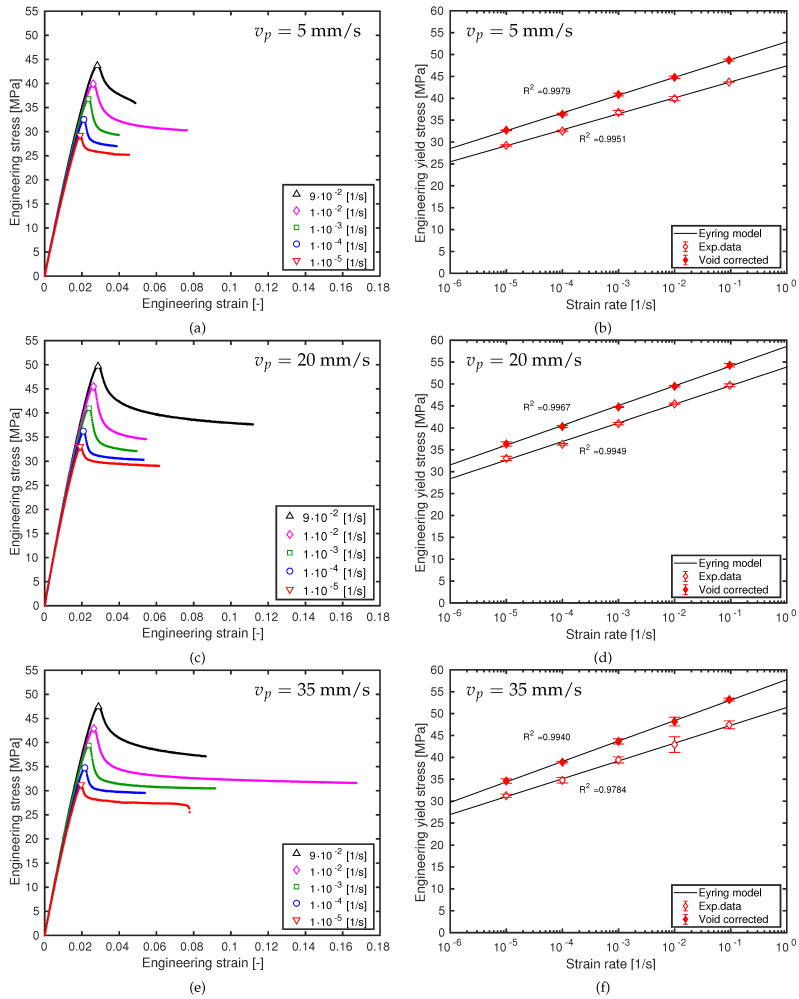
Engineering stress/strain response of ABS samples at 3 printing velocities. (**a**,**c**,**e**) stress as a function of strain. Symbols are experimental yield stress values; (**b**,**d**,**f**) yield stress and void corrected yield stress as a function of logarithmic strain rate. Symbols are experimental results, solid lines are model predictions. (**a**,**b**) $v_{p}=5$ mm/s; (**c**,**d**) $v_{p}=20$ mm/s; (**e**,**f**) $v_{p}=35$ mm/s.

The mechanical characterization results for the ME-AM processed samples at three different printing speeds are shown in Figure 2. The stress–strain curves are the average values of three tensile test results. The average engineering properties and their standard deviations are given in Table 2.

All ME-AM samples, independent of the printing speed, showed stress-whitening that started to appear right before the yield stress and emphasized with increasing strain. This phenomenon is related to crazing and is a standard observation for both injection molded [52,54] and ME-AM processed [9,23] ABS materials. Evidence of this phenomenon will be shown in the SEM fractography subsection. Stress-whitening, however, was non-uniform over the gauge section of the ME-AM test samples, as was also reported by Rodríguez et al. [9].

Ductile behavior was observed for all tensile test samples, in agreement with injection molded ABS [52,54] and ABS monofilament results [9,57]. However, ME-AM processed samples frequently show semi-ductile or even brittle behavior [9,23]. This indicates that the printing parameters used in this study are suitable to give adequate tensile properties. Generally speaking, Figure 2 shows that ductility slightly increases with printing speed. However, at the highest two strain rates, this does not completely hold up. It is assumed that local defects have a higher influence on failure behavior at higher strain rates, due to the fact that the material stiffens as strain rate is enhanced.

Another indication for proper printing parameters is given by the fact that the yield stress values measured in the present paper are above [8,9,57,58] or similar to [23,58] values measured previously on ME-AM processed ABS materials. Results are, however, slightly lower than for uniaxial tensile tests performed on samples that were injection-molded with a general-purpose ABS grade [52,54].

The elastic modulus for a single set of ME-AM samples stays almost constant, or slightly increases by 0.1 GPa with increasing strain rate. However, between the sets with different printing speeds, small variations can be detected. The set at $v_{p}=5$ mm/s has the lowest elastic modulus of *E* = 1.9–2.0 GPa, while samples fabricated at $v_{p}$ = 20 mm/s have values around 2.2 GPa. In between values of *E* = 2.0–2.1 GPa are seen at $v_{p}$ = 35 mm/s. Furthermore, and as is standard for viscoelastic materials, the strain at yield $\varepsilon_{y}$ rises for increasing strain rates $\dot{\varepsilon }$, in this case from 1.9% to 2.9% for all three sets.

The samples have an average apparent density $\rho_{app}$ between 0.93 and 0.95 g/cm${}^{3}$, in contrast to a material density of $\rho_{ref}=1.04$ g/cm${}^{3}$ as given by the filament producer. This agrees with sample porosities between 8.8% and 10.5%. As can be seen from Table 3, average apparent density and porosity for the sample sets fabricated at a print velocity of $v_{p}=20$ mm/s is slightly higher and lower, respectively, than for the other two sample sets. This set also manifests the higher mechanical properties (see Table 2). Possible reasons for this effect on mechanical properties will be given in the SEM fractography subsection.

The mechanical property results seem to be in agreement with the results shown by Abbott et al. [8]. Their high print speed ($v_{p}=50$ mm/s) negatively affected tensile strength, compared to their low print speed ($v_{p}=10$ mm/s). Furthermore, and although it is a carbon fiber-reinforced ABS material, Ning et al. [59] showed that an intermediate infill speed gave the best tensile strength performance. Nevertheless, also for carbon fiber-reinforced ABS, it is mostly the matrix material that determines the tensile strength properties [23], while the fibers govern stiffness.

As the results from Table 3 indicate, the various samples have different porosities. The interstitial voids in an ME-AM laminate composite structure, responsible for the sample’s porosity, logically have an impact on the stress–strain results. Hence, a compensation is applied to the yield stress in order to convert results for the voided structure into “solid” sample results by using Equation (3). This compensation is applied by using apparent density and yield stress values for every single sample separately. In this way, the effect of the printing velocity on the material behavior in a macroscopic sense can be determined. The *void corrected* yield stresses are shown in the images of Figure 2 on the right-hand side, represented by the solid symbols. These corrected values, by the way, are now in agreement with previously published yield stress values of injection-molded samples [52,54].

### 3.2. Analysis of Variance

To check consistency of the engineering yield stress experimental data and establish the level of statistical significance, an analysis of variance (ANOVA) was carried out. A linear interaction model was used for the analysis. The yield stress values were taken as the response, while logarithmic strain rate and print velocity were used as quantitative factors. The ANOVA results are given in Table 4.

If a *p*-value is less than 0.05, the related factor is significant within a 95% confidence level. Similarly, p<0.01 indicates that the factor is statistically significant at the 99% confidence interval. For p>0.05, there is a lack of fit to the response surface. The ANOVA results from Table 4 show that both strain rate and print velocity are statistically significant factors. The interactive term $\dot{\varepsilon }\times v_{p}$ turns out not to be of significance. This is in agreement with results from Van Erp et al. [33], who observed that the effect of strain rate and orientation on yield stress can be separated in a multiplicative way. It will be illustrated in the next subsection that polymer chain orientation is affected by the printing velocity.

**Figure 3:**
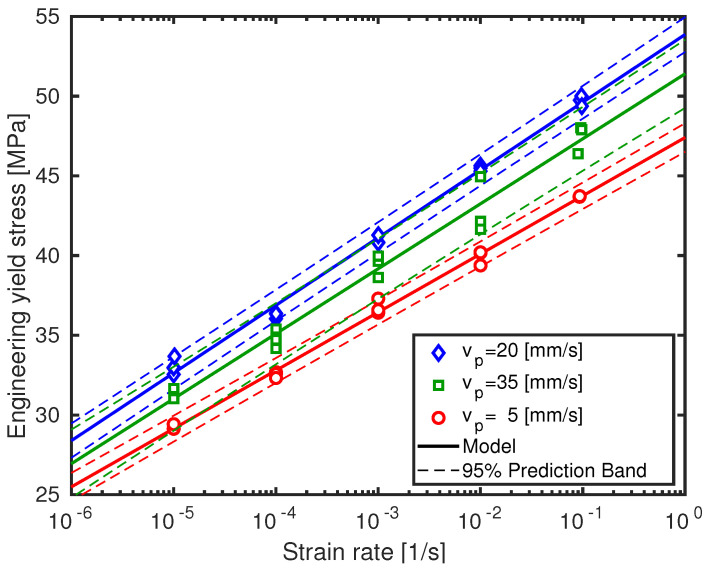
Engineering yield stresses and 95% prediction bands as a function of logarithmic strain rate for samples processed at three different printing velocities. Symbols are experimental results, solid lines are model predictions.

To show the dispersion of the engineering yield values (without void correction) and the influence of strain rate and print speed on these values, the separate values and the 95% prediction bands are displayed in Figure 3. It becomes clear from the figure that experimental dispersion augments as printing velocity increases. Although prediction bands overlap at the lower strain rate range, they start separating towards higher strain rates. In fact, none of the separate yield stress values fall within the 95% prediction bands of the other two printing velocities at the highest strain rate. This clearly indicates a meaningful effect of the print speed.

### 3.3. Polymer Chain Orientation and Stretch

The results for the polymer chain orientation and stretch via thermal shrinkage measurements are given in Table 5. All samples show a length contraction in the filament deposition direction and dimensional expansion in the perpendicular directions; moderate in the width and more pronounced in the height direction. This implies orientation and stretch of the polymer molecules in the deposition direction, as was also seen for material extrusion additively manufactured polylactide (PLA) samples [17]. Furthermore, a higher dimensional change indicates a higher degree of molecular orientation [9]. As the only parameter that has changed for the different sample sets is the print velocity $v_{p}$, it can be deduced from these results that infill speed is an ME-AM processing parameter responsible for a change in the resulting polymer chain orientation and stretch. Nevertheless, note that this does not mean that it is the *only* parameter having this effect. The estimated macroscopic plastic pre-strain $\varepsilon_{pre}$ for these samples, as given in Table 5, is calculated from the length shrinkage measurements.

Notice that dimensional length contraction for the feedstock filament is even higher than for the ME-AM processed samples. This implicates that the filament production process induces a high degree of polymer chain orientation and stretch for this highly elastic ABS material. Hence, it seems that, for ABS, the printing process reduces this production-induced orientation by relaxation of the polymer molecules, rather than imposing it. This was also observed by Rodríguez et al. [9]. From the tendencies shown in Table 5, it is concluded that the infill velocity affects polymer orientation and stretch. However, its effect is not straightforward, and will be elaborated on in the next subsection.

### 3.4. Strain-Dependent Activation Volume

As can be seen in the images of Figure 2 on the right-hand side, strain-rate sensitivity of the yield stress can be well captured using thermorheologically simple material behavior (Equation (6)) for all three printing velocities. Thus, also from these experimental results, there is no evidence that ABS materials are governed by two molecular deformation processes. However, each print speed does need its own rate constant ${\dot{\gamma }}_{0}$ and activation volume $V^{*}$ to correctly describe the experimental data. These model parameters are given in Table 3, and are lying in the same range as previously published results [9,23,52,54].

For all three print velocities, the coefficient of determination $R^{2}$ is close to unity. Print speed slightly lowers $R^{2}$, as higher speeds provoke higher experimental dispersion (see Figure 3). Notwithstanding, the coefficient of determination improves for the void corrected results. This indicates that an Eyring-type flow equation is able to correctly describe the thermorheologically simple behavior of this ME-AM processed ABS material as demonstrated by the strain-rate sensitivity of the yield stress data (Figure 2b,d,f).

**Figure 4:**
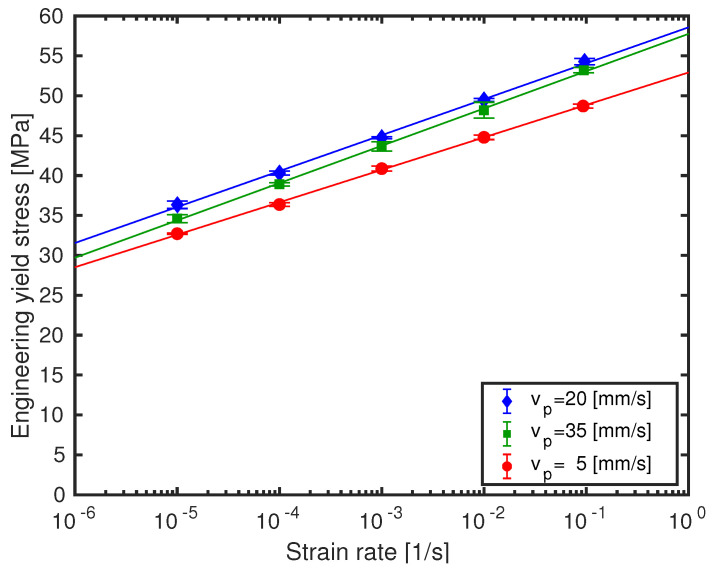
Void corrected engineering yield stress as a function of logarithmic strain rate for samples processed at three different printing velocities. Symbols are experimental results, solid lines are model predictions.

Table 3 shows a clear trend that the activation volume $V^{*}$ reduces with printing speed. The rate constant ${\dot{\gamma }}_{0}$, however, stays almost constant, within experimental error, with an increase at the highest print velocity. These effects are visually demonstrated in Figure 4, where the *void corrected* engineering yield stresses are shown as a function of the logarithmic strain rate for the three different printing speeds for better comparison. The strain-rate slope increases from 4.1 MPa/decade at print velocity $v_{p}=5$ mm/s, towards 4.5 MPa/decade at $v_{p}=20$ mm/s, while the highest printing speed $v_{p}=35$ mm/s exhibits 4.7 MPa/decade, which is due to the decreasing activation volume. Yield stresses for the ABSv35 sample set (printed at $v_{p}=35$ mm/s), though, manifest lower values than for set ABSv20 (i.e., the sample set printed at $v_{p}=20$ mm/s). Possible reasons for this will be given in the SEM fractography subsection. The lower values for ABSv35 result in an increment of its rate constant.

This last, rather surprising, effect where higher strain-rate sensitivity does not lead to higher yield stresses, is generally not seen in other research on oriented polymers [24,25,26,27,28,33,34]. As was mentioned in a previous research paper [17], it is assumed that the combination between the temperature (initial fast cooling combined with successive heating cycles [10,11,15,20]) and strain (shear effects in nozzle and during strand deposition [6,13,19]) profiles a material element experiences over time during ME-AM processing may be responsible for this effect. Those *time-dependent* profiles affect the (oblong) shape of the deposited strand, contact area and bond width and height, wetting and molecular diffusion at the interface driven by reptation, bond strength between adjacent and stacked strands, and orientation and stretch of the polymer chain [8,9,17,19]. This leads to spatially heterogeneous mechanical properties within a single sample, resulting in a certain macroscopic yield stress. For example, higher printing velocities provoke minimum temperatures to raise, maximum temperatures to decrease, and to reduce the time between peaks in the successive heating cycles [8,11], thus affecting the average temperature and the time the material stays below and above the glass transition temperature $T_{g}$. Above $T_{g}$, molecular chain mobility is significantly enhanced and the polymer molecule has an ability to relax, while, below $T_{g}$, the thermodynamic state of the material changes due to physical aging [16,53], resulting in increasing yield stresses. Furthermore, apart from the initial molecular orientation present in the filament feedstock material, shear effects may provoke some, temporary, extra molecular orientation, which depends completely on the available time and temperature. In addition, as was also mentioned in the introduction, printing parameters have an impact on these profiles. Hence, it is clear that the interplay between those two *time-dependent* profiles is complex during ME-AM processing and does not facilitate interpretation. It is also worth mentioning that these two profiles are significantly different from more conventional processing methods.

**Figure 5:**
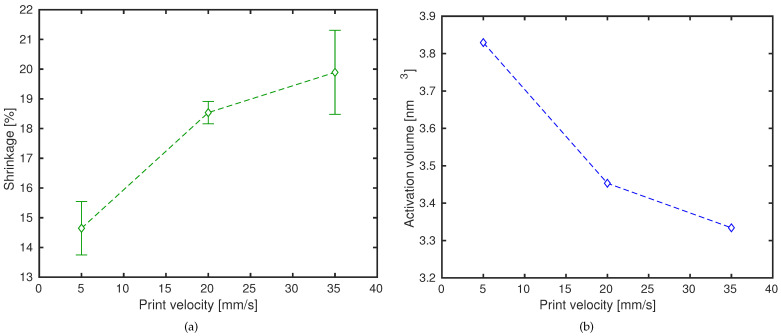
(**a**) dimensional length changes as a function of print velocity. Symbols are experimental results. Lines are a guide to the eye; (**b**) determined activation volume as a function of print velocity. Lines are a guide to the eye.

As was shown in the previous subsection, printing velocities have an influence on orientation and stretch (Table 5). However, as is shown in Figure 5a, there is no linear relation between the dimensional length contraction (a measure for molecular orientation) and the print speed. In fact, length shrinkage “slows down” as printing velocity increases. Likewise, the activation volume $V^{*}$ reduces with printing speed, but not in a linear way (see Figure 5b). The trend shown by both images in Figure 5 is, nevertheless, very similar.

**Figure 6:**
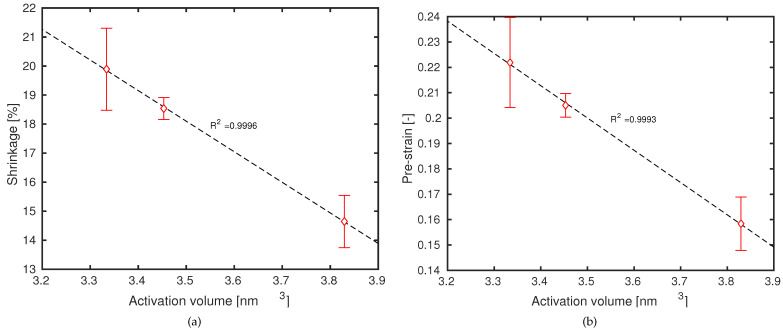
(**a**) dimensional length changes as a function of determined activation volume; (**b**) estimated pre-strain as a function of determined activation volume. Symbols are experimental results. Lines are a guide to the eye.

Thus, it is considered that the infill velocity parameter influences the time-temperature and time-strain profiles and, as a consequence, will lead to a resulting molecular orientation and stretch, i.e., a processing-induced pre-strain, in the final ME-AM sample. By now plotting the dimensional length contraction, or the estimated pre-strain $\varepsilon_{pre}$, as a function of the activation volume $V^{*}$ (see Figure 6), there appears to be a linear relation. Thus, it is suggested that ABS material exhibits a deformation-dependent activation volume, similar to what was seen for oriented iPP [33,34]. As was already mentioned by Senden et al. [34], a deformation-dependent activation volume does not necessarily apply to all polymers. Results for material extrusion additively manufactured polylactide, for instance, seem to demonstrate a deformation dependence of the rate constant [17], as was also found for pre-oriented PC [34].

Notwithstanding, at this point, it can not be ruled out that polymers show a strain-dependent activation volume in combination with a rate constant that also depends on deformation. Depending on the polymer and the strain-range, only one or the other may become visible during mechanical characterization. In the present study, only a narrow range of orientation and stretch was achieved with the printer settings used, leading to a rather limited range of estimated pre-strain ($\varepsilon_{pre}=0.15-0.23$). Even so, it seems unlikely that it is possible to extend that range much further by changing printer parameters. For a PLA polymer, using similar print velocity settings, significantly less molecular orientation and stretch was obtained [17]. Furthermore, mechanical characterization results were restricted to room temperature. Thus, the experimental range may be too limited to show both strain-dependent effects.

Other research on oriented polymers, using different processing techniques to accomplish molecular orientation, shows a more extended range of pre-deformation. Maximum (estimated) pre-strains reach $\varepsilon_{pre}=0.6$ for mechanical pre-orientation [34], $\varepsilon_{pre}=1.0$ in injection molding [36], $\varepsilon_{pre}=1.25$ in hydrostatic extrusion [27], and $\varepsilon_{pre}=1.8$ in hot drawing [33]. Generally, mechanical characterization was performed on a single test temperature in these studies. However, in none of the results, a combined activation volume/rate constant that depends on strain was observed. Nevertheless, these ranges may still not be enough, showing only either one of the deformation-dependent effects. Compare it, for example, with the thermorheologically simple behavior that polycarbonate commonly demonstrates for yield stress behavior as a function of strain rate [29]. Only for a wide enough range of strain rates and temperatures, PC starts to show thermorheologically complex behavior [43,60].

Therefore, to conclude if a polymer material shows a strain-dependent activation volume, a strain-dependent rate constant, or a combination of both, a wider range of molecular orientations (i.e., pre-strains) and temperatures is convenient. These aspects of material behavior should be taken into account while developing quantitative predictive numerical tools for ME-AM. These tools can help to design and manufacture safe, personalized, sustainable, and durable structural components using a relatively new and up-coming production technique.

### 3.5. SEM Fractography

**Figure 7:**
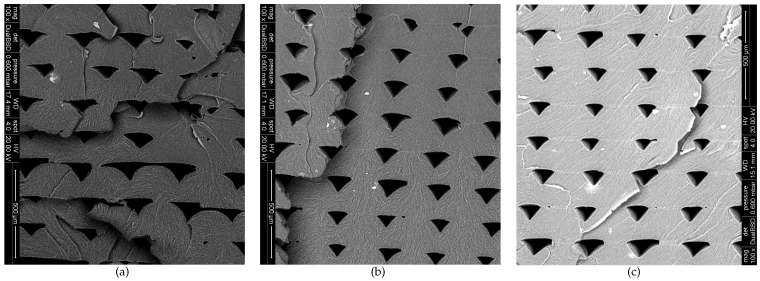
SEM micrographs of the ME-AM sample fracture cross-sections for 3 printing speeds. (**a**) $v_{p}=5$ mm/s; (**b**) $v_{p}=20$ mm/s; (**c**) $v_{p}=35$ mm/s.

In Figure 7, representative SEM images are shown corresponding to the fracture surfaces of the ME-AM ABS samples printed at the three different velocities. The individual arc-shaped strands and triangle-shaped inter-strand and inter-layer voids can be easily recognized for all three printing speeds. All three samples show local ductile tearing regions and small white lines corresponding to crazing as is typical for ABS materials [54]. Although not shown here, no significant differences were observed for samples that were characterized at different strain rates.

Figure 7a, corresponding to $v_{p}=5$ mm/s, demonstrates larger voids and less regular strand deposition, compared to the other two velocities. Furthermore, layer bond adhesion is also significantly worse, with occasionally a very low adhesion width or even an absence of adhesion between strands in adjacent layers. Adhesion height between adjacent strands in the same layer is also more irregular than for the other two samples. These effects are assumed to be due to the complex interplay between the temperature and strain profiles a material element experiences over time during ME-AM processing, and are thought to be responsible for the lower apparent density and yield stresses.

In Figure 7a,b, weld lines become visible between layers, and at times also between adjacent strands in the same layer. For $v_{p}=20$ mm/s (Figure 7b), these weld lines are not seen at the bottom layers, but become visible and get more pronounced towards the top layers. On the contrary, in Figure 7b, these lines are present in all layers. Such weld lines are due to incomplete diffusion of the material from adjacent strands and, as a consequence, negatively affects the inter-strand bonding and, thus, macroscopic properties. Although it can not be confirmed here, it has previously been indicated that molecular orientation hinders molecular diffusion at the interface [19]. Again, the complex interplay of the temperature and strain profiles plays a role. It is suggested that the amount of visible weld-lines is responsible for the lower yield stresses for $v_{p}=35$ mm/s.

**Figure 8:**
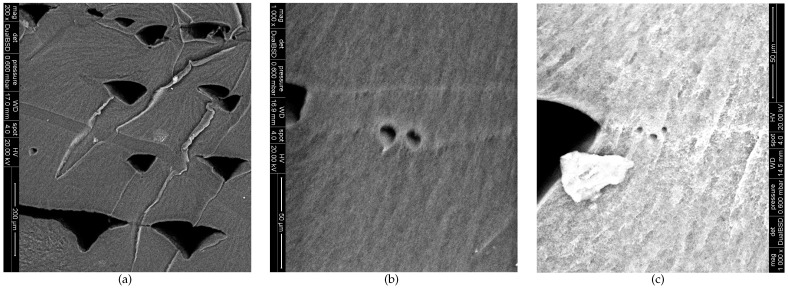
Detailed SEM micrographs of the ME-AM sample fracture cross-sections for 3 printing speeds. (**a**) $v_{p}=5$ mm/s; (**b**) $v_{p}=20$ mm/s; (**c**) $v_{p}=35$ mm/s.

More detailed images at higher amplifications of the fracture surfaces are demonstrated in Figure 8. As the welding lines are not visible for the lowest printing speed, an image of a zone with local ductile tearing is shown for $v_{p}=5$ mm/s (Figure 8a). This image again exhibits the irregularity of the inter-strand voids for this printing speed.

Figure 8b,c display a close-up of a welding line for the other two printing speeds. Both images show that, in the welding lines, small voids are present that are difficult to detect at a lower amplification. This is an effect seen for most of the weld lines present in the samples. It is at present unclear if these voids already exist before fracture, or if they are an effect of the fracture process.

## 4. Conclusions

Strain-rate sensitivity of the yield stresses for Acrylonitrile-Butadiene-Styrene (ABS) tensile samples processed via material extrusion additive manufacturing (ME-AM) was measured and analyzed. Attention was focused on the relation between printing velocity and resulting molecular orientation and stretch, and how this influences material behavior such as the strain-rate dependence of the yield stress. Apparent densities of ME-AM processed test samples were measured and taken into account for calculating *void corrected* yield stresses. In this way, yield stresses are compensated for the interstitial voids, i.e., porosities, that are present in the ME-AM laminate composite structure and the effect of the processing step on the material behavior can be determined. Evaluated apparent densities for the produced ABS samples agree with sample porosities of around 10%.

All ME-AM samples showed ductile behavior in the tensile tests. In addition, yield stress values were close to previous published results for injection-molded specimen [52,54], especially when compensated for the interstitial voids. This indicates that the printing parameters used in this study are suitable to give adequate tensile properties. Furthermore, the results illustrate that, with ME-AM techniques, mechanical properties can be achieved that are close to, although slightly below, bulk properties.

Three different infill velocities, i.e., printing speeds, were used to generate ME-AM tensile test specimens. SEM fractography images revealed that the lowest print velocity ($v_{p}=5$ mm/s) resulted in a less regular strand deposition and larger inter-strand and inter-layer triangle-shaped voids. Higher infill speeds led to the appearance of visible weld lines between adjacent layers and strands. The amount of weld lines increased as the printing velocity increased. These effects are thought to influence the yield stress values.

Higher printing velocities resulted in samples with higher molecular orientation and stretch, as determined from thermal shrinkage measurements. However, the ABS filament, as applied in the present study, showed an even higher molecular orientation. Hence, the ME-AM process reduces the filament production-induced orientation, rather than imposing it. Rodríguez et al. [9] came to the same conclusion in their work.

Thus, the effect of the printing velocity on the molecular orientation and stretch is not straightforward and deriving a direct relation seems complicated. Previous research [6,11,13,15,17,20], however, showed the importance of temperature and strain profiles on resultant properties. Investigating the relation between printing parameters and properties using intermediate data such as the time-temperature and time-strain profiles is, therefore, a recommended strategy.

The ABS material used here manifests thermorheologically simple behavior. A single molecular relaxation (α) mechanism is adequately able to capture the deformation behavior over the measured strain-rate range. This is in agreement with other previously published research on distinct ABS materials [9,23,52,54,55,56]. The Eyring-type flow equation is able to correctly describe the strain-rate sensitivity of the yield stress. However, each print speed does need its own rate constant ${\dot{\gamma }}_{0}$ and activation volume $V^{*}$ to correctly describe the experimental data.

The changing activation volume $V^{*}$, as a result of a varying print velocity, seems to scale linearly with the molecular orientation, as captured in an estimated processing-induced pre-strain $\varepsilon_{pre}$. Therefore, it is believed that ME-AM processed ABS shows a deformation-dependent activation volume, similar to what was shown for oriented isotactic polypropylene [33,34].

From an engineering design point of view, it is then desirable to include this deformation- dependent strain-rate sensitivity of the yield stress into quantitative predictive numerical tools. Such tools are important to determine up-front a structural application’s short- and long-term ultimate failure behavior (impact, creep, fatigue). For instance, to design and manufacture personalized, safe, sustainable, and durable biomechanical utilities for elderly and disabled people. The experimental results demonstrated in the present research is initial work that can help in this complex modeling task.

## Figures and Tables

**Table 1:** Processing parameters used to manufacture ME-AM tensile samples.

Processing Parameter	Value
Nozzle diameter [mm]	0.40
Extrusion width [mm]	0.30
Layer height [mm]	0.20
Number of perimeters	2
Infill pattern	Rectilinear
Fill percentage	100%
Outline overlap	80%
Extrusion temperature [${}^{\circ }$C]	230
Bed temperature $T_{b}$ [${}^{\circ }$C]	100
Infill orientation angle $\alpha_{or}$	$0^{\circ }$
Printing speed $v_{p}$ [mm/s]	5, 20, 35

**Table 2:** Average measured engineering mechanical properties for ME-AM samples. Standard deviations are indicated between brackets.

Sample	$\dot{\varepsilon }$	*E*		$\sigma_{y}$		$\varepsilon_{y}$		$\varepsilon_{b}$	
Nomenclature	[1/s]	[MPa]		[MPa]		[%]		[%]	
	$1\times 10^{-5}$	1886	(60)	29.24	(0.16)	1.90	(0.04)	4.53	(0.45)
	$1\times 10^{-4}$	1918	(9)	32.52	(0.18)	2.10	(0.01)	3.86	(0.93)
ABSv05	$1\times 10^{-3}$	1954	(29)	36.76	(0.47)	2.36	(0.04)	3.98	(0.86)
	$1\times 10^{-2}$	1978	(26)	39.92	(0.47)	2.61	(0.01)	7.63	(2.85)
	$9\times 10^{-2}$	1949	(12)	43.71	(0.03)	2.83	(0.03)	4.87	(0.20)
	$1\times 10^{-5}$	2192	(79)	33.04	(0.47)	1.91	(0.03)	6.14	(1.73)
	$1\times 10^{-4}$	2192	(38)	36.25	(0.19)	2.07	(0.02)	5.31	(2.80)
ABSv20	$1\times 10^{-3}$	2208	(5)	40.98	(0.26)	2.39	(0.01)	4.93	(2.51)
	$1\times 10^{-2}$	2202	(8)	45.47	(0.17)	2.62	(0.01)	5.44	(4.58)
	$9\times 10^{-2}$	2168	(47)	49.71	(0.32)	2.86	(0.05)	11.18	(5.62)
	$1\times 10^{-5}$	2018	(12)	31.24	(0.37)	1.95	(0.03)	7.78	(0.20)
	$1\times 10^{-4}$	2025	(22)	34.76	(0.61)	2.15	(0.05)	5.38	(0.62)
ABSv35	$1\times 10^{-3}$	2101	(53)	39.41	(0.70)	2.38	(0.04)	9.15	(3.49)
	$1\times 10^{-2}$	2084	(76)	42.91	(1.78)	2.64	(0.05)	16.74	(4.94)
	$9\times 10^{-2}$	2109	(44)	47.44	(0.90)	2.89	(0.02)	8.65	(5.97)

**Table 3:** Average apparent density, porosity, and model parameters used to describe the yield behavior of the ME-AM samples. Standard deviations are indicated between brackets.

Sample	${\bar{\rho }}_{app}$		Porosity		$V^{*}$	${\dot{\gamma }}_{0}$
Nomenclature	[$g/{cm}^{3}$]		[%]		[${nm}^{3}$]	[1/s]
ABSv05	0.93	(0.01)	10.50	(0.49)	3.83	$1.19\times 10^{33}$
ABSv20	0.95	(0.01)	8.84	(0.83)	3.45	$1.24\times 10^{33}$
ABSv35	0.93	(0.01)	10.37	(1.15)	3.33	$5.10\times 10^{33}$

**Table 4:** Analysis of Variance (ANOVA) results of an interaction model for the engineering yield stress versus logarithmic strain rate and print velocity.

Source	Degree of Freedom	Sum of Squares	Mean Square	F-Value	*p*-Value
Strain rate $\dot{\varepsilon }$	4	1387.85	346.962	857.99	0
Print velocity $v_{p}$	2	159.36	79.679	197.04	0
$\dot{\varepsilon }\times v_{p}$	8	6.43	0.804	1.99	0.0827
Error	30	12.13	0.404		
Total	44	1620.96			

**Table 5:** Average dimensional changes of ME-AM sample sets after a thermal treatment at 130 ${}^{\circ }$C for over six hours. Expansion: +; Contraction: −. Standard deviations are indicated between brackets.

Sample	Length		Width		Height		Pre-Strain $\varepsilon_{\mathrm{pre}}$	
Nomenclature	[%]		[%]		[%]		[-]	
Filament	−30.5	(2.8)	+20.6	(3.5)	+20.6	(3.5)	0.364	(0.041)
ABSv05	−14.7	(0.9)	+0.9	(0.7)	+17.9	(2.7)	0.158	(0.011)
ABSv20	−18.5	(0.4)	+2.8	(1.0)	+24.8	(2.0)	0.205	(0.005)
ABSv35	−19.9	(1.4)	+3.6	(1.9)	+26.7	(4.3)	0.222	(0.018)
